# Prevalence, Impact, and Management Practice of Dysmenorrhea among University of Gondar Students, Northwestern Ethiopia: A Cross-Sectional Study

**DOI:** 10.1155/2017/3208276

**Published:** 2017-05-14

**Authors:** Minaleshewa Biruk Gebeyehu, Abebe Basazn Mekuria, Yonas Getaye Tefera, Dagmawi Abate Andarge, Yabsira Belayneh Debay, Geremew Sokile Bejiga, Begashaw Melaku Gebresillassie

**Affiliations:** ^1^Department of Clinical Pharmacy, School of Pharmacy, University of Gondar, Gondar, Ethiopia; ^2^Department of Pharmacology, School of Pharmacy, University of Gondar, Gondar, Ethiopia

## Abstract

**Background:**

Dysmenorrhea is an important health problem of adolescents in school, as well as health practitioners, that badly affects the daily activities and quality of life. The aim of this study was to measure the prevalence of dysmenorrhea and assess its management practice among University of Gondar students.

**Methods:**

A cross-sectional study was done from April 06 to May 08, 2016, on female students of University of Gondar. Descriptive and binary logistic regression analyses were used to describe and assess the association between different variables.

**Results:**

More than two-thirds (75.3%) of the respondents were nonmedical students and the prevalence of dysmenorrhea was 77.6%. About half (50.6%) of the participants reported that they have a family history of dysmenorrhea and experienced continuous type of pain (53%) which lasts 1-2 days (47.8%). Abdominal spasm (70.4%), back pain (69.7%) fatigue, and weakness (63.5%) were the most commonly experienced dysmenorrhea symptoms. More than half (63%) of the respondents had encountered social withdrawal and decrease in academic performance (51.4%). More than two-thirds (63.8%) of the respondents use home remedies as a primary management option. Ibuprofen and diclofenac were the most commonly used medications to manage dysmenorrhea.

**Conclusions:**

The present study revealed that high proportion of University of Gondar female students had dysmenorrhea. Findings suggest the need for educating adolescent girls on appropriate and effective management of dysmenorrhea.

## 1. Introduction

Menstrual period is a natural phenomenon which occurs throughout the reproductive years of every woman. Most females experience certain degree of pain and distress during their menstruation period [1]. Dysmenorrhea is a painful/cramping sensation in the lower abdomen often accompanied by other biological symptoms including dizziness, fatigue, sweating, backache, headache, nausea, vomiting, and diarrhea all occurring just before or during the menstruation.

Dysmenorrhea may be categorized into two types as primary and secondary. Primary dysmenorrhea is defined as painful menses among females with normal pelvic anatomy, frequently beginning during adolescence. It is observed only in ovulatory cycles, frequently emerging within 6 to 12 months after menarche with no pathology or organic basis. Secondary dysmenorrhea is a menstrual pain associated with underlying pathology and its onset might be years after menarche [2].

Due to its importance, different treatments including pharmacological and nonpharmacological treatment approaches such as taking nonsteroidal anti-inflammatory drugs (NSAIDS), herbal, dietary therapies, yoga, meditation, and acupuncture have been used to lessen the effects of dysmenorrhea [3].

According to Ethiopian standard treatment guideline, dysmenorrhea occurs in about 50% of menstruating women. Dysmenorrhea in some years following menarche is usually primary, but the secondary characteristically occurs many years after menarche.

Primary dysmenorrhea is extremely common, especially among adolescents. As many as 90% of adolescent females and above 50% of menstruating women worldwide report suffering from it, with 10–20% of them describing their hurt as severe and distressing [4].

Dysmenorrhea is a cause of frequent short-term work and school absenteeism in women of reproductive age. Approximately 10–15% of females experience monthly menstrual pain severe enough to stop normal daily functions at work, home, or school [2].

Even though primary dysmenorrhea is not a real threat of life but can affect the quality of females' life and in case of severity it might lead to disability and inefficiency. Moreover, dysmenorrhea can cause mental problems in some of the females resulting in their loneliness and reduced participation in different social activities.

In adolescents, moderate to severe pain that affects lifestyle and does not respond to pharmacological treatment requires professional attention and appropriate diagnosis of possible underlying pelvic disease. The exact prevalence of dysmenorrhea is difficult to determine because of variety of diagnostic criteria and the subjective feature of the symptoms. In many countries, primary dysmenorrhea is the principal cause of recurrent short-term work and school absenteeism in young girls and women [3].

We could not found a study which was done on the prevalence and management of dysmenorrhea and this study has significant importance to determine and explore the prevalence, impact, and treatment practice of dysmenorrhea. Moreover, the findings might have significant impact in decreasing risks associated with self-management of dysmenorrhea.

## 2. Materials and Methods

### 2.1. Study Setting and Period

An institution-based cross-sectional study was employed to assess the prevalence, impact, and management practice of dysmenorrhea among female students of University of Gondar (UOG) from April 06–May 08, 2016. UOG is one of the oldest and most well established higher education institutions in the country and located 738 km northwest of Addis Ababa which is a capital city of Ethiopia. It has five campuses, namely, Maraki, Atse Tewodros, Atse Fasil, College of Medicine and Health Sciences (CMHS), and Meles Zenawi. Currently, through nine academic units; College of Medicine and Health Sciences, Business and Economics, Natural and Computational Sciences, and Social Sciences and Humanities; Faculty of Veterinary Medicine and Agriculture; Schools of Law, Technology, and Education, it offers 64 postgraduate and 56 undergraduate programs in regular, extension, summer, and distance programs.

### 2.2. Study Population

The source population includes all female students currently learning at University of Gondar, whereas the study population includes female students who were available during the data collection period.

### 2.3. Sample Size Determination and Sampling Procedure

The sample size was calculated using a single mean formula [5]:

(1) $$\begin{array}{c} n=\frac{z^{2}p\left(1-p\right)}{d^{2}}, \end{array}$$

where *n* is the sample size required; *d* is marginal error of 5% (*d* = 0.05); *z* is the degree of accuracy required at 95% confidence level = 1.96; *p* is the proportion of dysmenorrhea occurrence among female University of Gondar students (0.5 [50%]).

Using the aforementioned formula,

(2) ni1.9620.51−0.50.052=3.84160.250.0025=0.96040.0025=385.

Since the sample was taken from source population of 6211 female students, which is <10,000, the final sample size was calculated after using correction factor:

(3) nf=ni1+ni/N,nf=3851+3856211=3851.0619=362.

After 10% of the calculated sample size was added for possible nonresponse, the final sample size became 400 female students.

**Figure 1:**
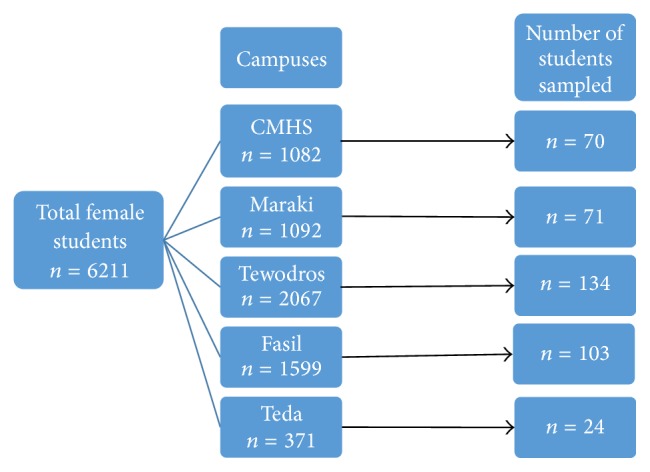
Flowchart of sampling procedure (selected from each campus using simple random sampling technique).

Out of 6211 female students currently learning at University of Gondar, 400 students from all five campuses were drawn by employing stratified and simple random sampling technique. The required number of samples from each campus was determined by multiplying the ratio of total female students in each campus to total number of female students in the university (6211) with the calculated final sample size (400). Following determination of number of samples to be taken from each campus, using simple random sampling technique, the required samples were selected (Figure 1).

### 2.4. Study Variables

The independent variables include age, sex, family history, ethnicity, monthly income, knowledge, duration of menarche, and menstrual irregularity. The dependent variables were the prevalence, impact, and management practice of dysmenorrhea.

### 2.5. Data Collection and Management

The data collection tool used in the study was adopted from previous studies and prepared in English. This was translated to Amharic local language and then back to English in order to ensure that the translated version gives proper meaning. The questionnaire has three major parts which focused on sociodemographic characteristics, menstruation characteristics and its impact, and management practice of dysmenorrhea. The data collection instrument was pretested on 20 students who were not included in the final analysis and relevant modifications were instituted before the commencement of actual data collection. The data were collected by eight principal investigators through interviewer-administered questionnaires and face-to-face interview by explaining the questions for those who were unable to understand. The investigators who collected the data were properly trained on the instrument and ways of approaching the students and securing their permission for interview prior to the data collection process.

### 2.6. Data Entry and Analysis

The collected data using quantitative method were cleaned, entered into, and analyzed using IBM SPSS version 20.0. In the study, sociodemographic characteristics, prevalence, impact, and management practice of dysmenorrhea were described using frequencies, percentage, and mean and standard deviation. Binary logistic regression analysis was employed to determine the association between different variables, and *P* values less than 0.05 and 95% confidence interval (CI) were used as cutoff points for determining statistical significance of associations among different variables.

### 2.7. Ethical Consideration

This study was conducted after ethical clearance was gained from research and ethics review committee of School of Pharmacy. Verbal informed consent was obtained and each of the participants was provided with explanations on the purpose of the study. They were also informed that participation was voluntary and they could withdraw from the study at any stage if they desired.

### 2.8. Operational Definitions

Home remedies included coffee, tea, coca, cold bath, massage, heat compression, and their combination.

Nondrug users are participants who do not use medication for their dysmenorrheal pain.

Antipyretic that was used is paracetamol.

NSAIDS that were used are ibuprofen and diclofenac.

Opioids that were used included tramadol.

Contraceptives that were used were oral contraceptive pills.

## 3. Results

### 3.1. Sociodemographic Characteristics of Respondents

**Figure 2:**
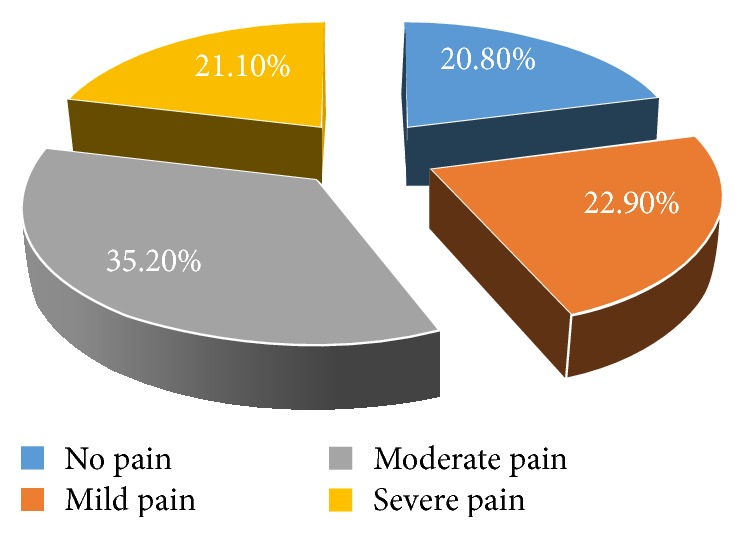
Degree of pain among female students of UOG, Gondar, 2016.

**Figure 3:**
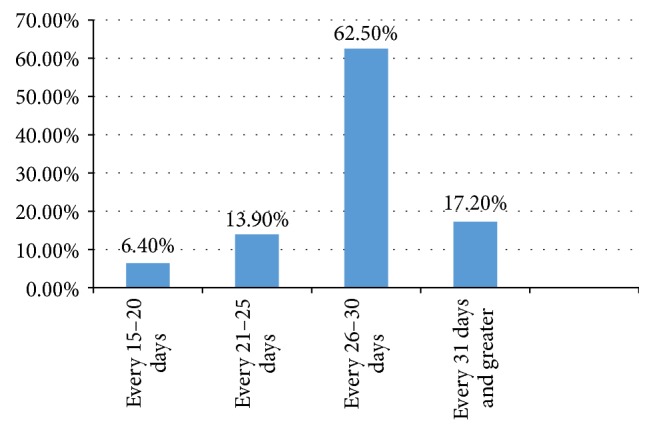
Length of menstruation cycle among female students of UOG, Gondar, 2016.

**Figure 4:**
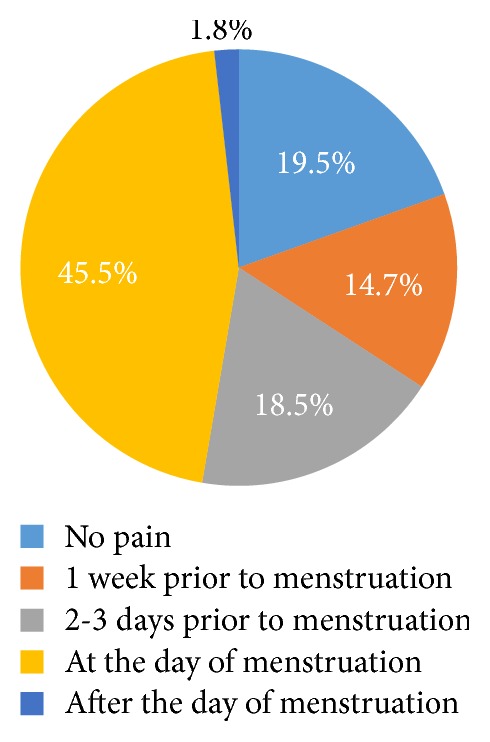
Onset of dysmenorrhea among female students of UOG, Gondar, 2016.

In this study, out of the total interview guides/questionnaires of sample of 400 students who were interviewed, 389 were included in the analysis and 11 encounters were excluded due to incompleteness making the response rate 97.3%. The average age of study respondents was 21 years. More than two-thirds (75.3%) of the study participants were nonmedical students and age at which they started menstruating was 12–14 years. About half (50.6%) of the respondents reported that they have a family history of dysmenorrhea and the pain starts to occur at the time of menstruation for 45.5% of the respondents (Figure 4). Along with this, about (35.2%) experienced moderate and continuous type of pain (53%) which lasts for 1-2 days (47.8%) (Figure 2). In more than two-thirds (62.%) of the respondents, their menstruation cycle length ranged within 26–30 days with the duration of flow being 3-4 days (Figure 3). The most frequently experienced dysmenorrhea symptoms reported were abdominal spasm (70.4%), back pain (69.7%) fatigue, and weakness (63.5%), respectively (Table 1).

### 3.2. Impact and Management Practice of Dysmenorrhea

More than two-thirds (63%) of the respondents reported that they had encountered social withdrawal and decrease in academic performance (51.4%). More than one-third (40.9%) of the respondents experienced restrictions from day-to-day activities during their menstrual period and associated with this about 31.1% were absent from class and reported poor concentration (43.4%). Nearly half (42.7%) of the respondents reported they had decreased appetite and altered sleeping pattern.

Out of all the respondents, only 8% of them performed physical exercises and about two-thirds (60.9%) of them were using home remedies as a nonpharmacological treatment option of dysmenorrhea, whereas only 16.2% the respondents consult health professionals about their dysmenorrheal condition. About 36.3% of them were using medications to manage their dysmenorrheal pain and ibuprofen (12.6%), diclofenac (6.9%), and paracetamol (5.4%) were the most frequently used medications, whereas coffee, tea, and Coca-Cola (34.4%), and heat therapy (3.9%) were among the most frequently used home remedies to manage their illness.

Out of those respondents who were using medications, the majority (82.3%) of them took their medications at the time of menses (75.9%) 1-2 times per day in PO rout of administration (89.4%) for 1-2 days (61%) (Table 2).

### 3.3. Respondents' Knowledge about Medication

In this study, out of 389 respondents, only 15.4% and 12.6% of them knew about the precautions and warnings and contraindications of the drugs they were taking, respectively. Regarding dosage of the medications, only 17.7% knew the recommended maximum daily dose of the drugs. Along with this, also only 12.6% of the respondents knew contraindications of the drugs they were taking to manage their pain. Similarly, the majority of the respondents did not know about the adverse effect associated with the drugs they were taking (Table 2).

### 3.4. Predictors of Dysmenorrhea, Medication, and Home Remedy Use

In logistic regression analysis, family history of dysmenorrhea and duration of menstrual blood flow were found to have independent determining factors for the probability of dysmenorrhea to occur. Students with five and more days of menstrual blood flow (AOR = 0.292, 95% CI = 0.109–0.783) were found to be 70.8% less likely not to have dysmenorrhea taking 1-2 days of menstrual blood flow as a reference. On the other hand, being health science students and severity of menstrual pain were statistically significant predictors of medication use. Students from medical (health) faculty (AOR = 0.228, CI = 0126–0.413) were found to be 77.2% less likely not to use medications (Table 3).

## 4. Discussion

Among the 389 participants, the overall prevalence of dysmenorrhea among University of Gondar students was found to be 77.6% which was in agreement with the prevalence rate reported by study from Saveetha University, Universitaria Policlinico of Modena, and Private University in Ogun State, which were 70.6%, 84.1%, and 78.1% for the same question, respectively [2, 6, 7].

In this study, only 16.2% of the participants consulted health professionals about their menstrual pain. More than half (53.7%) of them endure their pain and only 36.5% of them uses medication. Regarding these, a similar study done in Nigeria showed that only 7.9% consulted a healthcare professionals and nearly half (46.3%) of participants endured dysmenorrhea, along with this only 29.3% of the participants managed themselves with over-the-counter medications [2]. This might have resulted from variation of baseline knowledge, attitude, and management practice of dysmenorrhea between these population groups.

About half of the students (50.6%) have family history of dysmenorrhea, out of which about 81.3% experience pain during their menstruation. This implies that family history and irregular cycle of menstruation can be taken as risk factors to experience dysmenorrhea.

Among the impacts of dysmenorrhea, absenteeism from school, poor concentration, sleep disorder depression, and behavioral change like social withdrawal and restriction from daily activities were the most frequent in this study. Regarding these, similar results were reported from Nigeria, which shows that there has been an increased number of absenteeism and decreased normal daily functions [2]. These results revealed that during dysmenorrhea students will face a tremendous impact in their educational outcomes and daily activities.

In this study, about 8% of the students carried out physical exercise as a measure to control their dysmenorrheal illness. Regarding this, a similar study conducted in Iran supports use of physical activity had positive impact on the most of primary dysmenorrhea symptoms [3, 8]. Unfortunately, none of the respondents from Nigeria reported engaging in physical exercise in order to manage their menstrual pain, rather about 77.2% restricted physical activity because of the severity of the pain [2].

The present study showed that most of the participants were using home remedies as a nonpharmacologic treatment option for dysmenorrheal pain. As the degree of pain increases, the use of home remedies increases. Along with this, as the pain increases, the students were using more than one type of home remedies at a time. These findings were in agreement with systematic review report from Australia among Chinese, which shows that university students were using Chinese traditional medicine as a nonpharmacological treatment [9].

About 22.9% of students who were using medications were from nonmedical faculty and they were not aware of the drugs precaution, contraindication, maximum dose, adverse effect, dose, frequency, and duration. This may lead to severe health related complications associated with misuse of drugs. NSAIDs like ibuprofen could result in gastrointestinal bleeding and worsen existing case of peptic ulcer disease [10].

Findings from this study also showed that self-medication practice was higher among students from medical (health) faculty as compared to students from nonmedical faculty. This finding is in contrast with the report from a study conducted in Hong Kong, which showed that more than half of the self-medicated women with dysmenorrhea were particularly nonmedical students [11]. This might be due to lack of medical knowledge associated with the illness and poor access to medications among nonmedical students.

In binary logistic regression analysis, family history and duration of menstrual blood flow were found to have independent determining factors for the probability of dysmenorrhea to occur. Students with five and more days of menstrual blood flow are found to be 70.8% less likely not to have dysmenorrhea, taking 1-2 days of menstrual blood flow as a reference. On the other hand, being health science students and severity of dysmenorrheal pain were statistically significant predictors of medication use. Students from medical (health) faculty and who have severe pain were more likely to use medications. The possible explanation might be that, among nonmedical students, there was a negative attitude towards dysmenorrhea, as they thought that dysmenorrhea is mostly considered as a normal part of female menstrual cycle and a burden every woman must bear, and knowledge about the available treatment approach is inadequate.

### 4.1. Limitation of the Study

The participants were selected from only one educational institution which limits the generalizability of the result to other settings. The self-reporting nature of the present study might have resulted in a recall bias and overreporting of the condition. This may have had an impact on the stated prevalence of the illness. Several factors that might affect menstrual outcome were not considered, including smoking, obesity, and stress. This cross-sectional study may serve as an insight for further studies to be conducted on this area that should adopt more rigorous designs to address the issue.

## 5. Conclusions

In this study, the overall prevalence of dysmenorrhea among University of Gondar students was found to be high. About half of the participants stated that they have a family history of dysmenorrhea and the pain starts to occur during the time of menstruation. Along with this, a large number of them experienced moderate and continuous type of pain which lasts for 1-2 days. More than two-thirds of the participants also stated that they had encountered social withdrawal and decrease in academic performance associated with this pain.

About one-third of the participants were using medications to control their dysmenorrheal pain. Along with this, the majority of the participants did not know about the adverse effect associated with the drugs they were taking. In logistic regression analysis, family history, degree of pain, and duration of menstrual blood flow were found to have independent determining factors for the occurrence of dysmenorrhea, home remedy, and medication use. Based on these findings, education on the appropriate management of dysmenorrhea should be given to students, parents, and hostel administrators in order to address the reproductive health needs of the female students.

## Figures and Tables

**Table 1:** Sociodemographic and menstrual characteristics of respondents, Gondar, 2016.

Variables	Frequency	Percent
*Year of study*		
First	114	29.3
Second	137	35.2
Third	77	19.8
Fourth	42	10.8
Fifth	16	4.1
Sixth	3	0.8
*Religion of the respondents*		
Muslim	21	5.4
Protestant	52	13.4
Catholic	2	0.5
Orthodox	313	80.5
*Family income*		
<500	128	32.9
500–1000	177	45.5
1001–2000	78	20.1
>2000	6	1.5
*Faculty*		
Medical	96	24.7
Nonmedical	293	75.3
*Age at menarche*		
9–11 years	22	5.7
12–14 years	230	59.1
15–17 years	137	35.2
*Experience dysmenorrhea?*		
Yes	302	76.6
No	87	24.4
*Family history of dysmenorrhea*		
Yes	197	50.6
No	192	49.4
*Duration of bleeding*		
1-2 days	186	47.8
3-4 day	96	24.7
5 and more days	31	8.0
*Type of pain*		
Continuous pain	108	27.8%
Intermittent pain	206	53%

**Table 2:** Types of medications used, knowledge about drugs, and medication practice of the respondents, Gondar, 2016.

Type of drugs used	Frequency	Percent
Nondrug users	248	63.8
Antipyretic	21	5.4
NSAIDS	91	23.4
Opioids	3	.8
Contraceptives	5	1.3
Antipyretic, NSAIDS, opioids, and oral contraceptives	3	8
NSAIDS and opioids	6	6
Antipyretic and NSAIDS	7	9
NSAIDS and contraceptives	3	.8
Antipyretic, NSAIDS, and opioids	2	5
*Route of administration*		
Nondrug users	248	63.8
PO route	126	32.4
IM (IV) route	7	1.8
PO and IM (IV)	8	2.1
*Frequency of drug used per day*		
Nondrug users	248	63.8
1-2 times per day	116	29.8
3-4 times per day	8	2.1
PRN (when needed)	17	4.4
*Initiation of medications*		
Nondrug users	248	63.8
One week prior to menarche	6	1.5
2-3 days before menses	16	4.1
At menarche	107	27.5
After menarche	12	3.1
*Duration of drug use*		
Nondrug users	248	63.8
1-2 days	86	22.1
3-4 days	13	3.3
PRN (when needed)	42	10.8
*Knowledge on the precaution/warning of the drug used*		
Yes	60	15.4
No	329	84.6
*Knowledge on the contraindication of the medication used*		
Yes	49	12.6
No	340	87.4
*Knowledge on the maximum recommended dose of the drug used*		
Yes	69	17.7
No	320	82.3
*Knowledge on the risk and adverse effect of the drug used*		
Yes	67	17.2
No	322	82.8

**Table 3:** Predictors of dysmenorrhea, medication, and home remedy use, Gondar, 2016.

Variables			COR (95% CI)	AOR (95% CI)	*P* value
	
	*Dysmenorrhea* (*n* = 389)				
	Yes (%)	No (%)			
*Family history of dysmenorrhea*					0.000^**∗**^
No	123 (31.6)	69 (17.7)	1	1	
Yes	179 (46.0)	18 (4.6)	0.179 (0.102–0.316)	0.192 (0.108–0.341)	
*Duration of menstrual flow (bleeding)*					0.044^**∗**^
1-2 days	16 (4.1)	12 (3.1)	1	1	
3-4 days	197 (50.6)	61 (15.7)	0.413 (0.097–0.683)	0.522 (0.224–1.218)	
5 and more days	89 (22.9)	14 (3.6)	0.210 (0.0609–0.321)	0.292 (0.109–0.783)	

	*Medication use (n* = 389)				
	Yes (%)	No (%)			
*Faculty*					0.000^**∗**^
Nonmedical	89 (22.9)	204 (52.4)	1	1	
Medical (health)	53 (13.6)	43 (11.1)	0.354 (0.221–0.568)	0.228 (0126–0.413)	
*Degree of menstrual pain*					0.002^**∗**^
No pain	2 (0.5)	79 (20.3)	1	1	
Mild pain	19 (4.9)	70 (18.0)	0.093 (0.021–0.415)	0.097 (0.021–0.439)	
Moderate pain	64 (16.5)	73 (18.8)	0.029 (0.007–0.122)	0.025 (0.006–0.103)	
Sever pain	57 (14.7)	25 (6.4)	0.011 (0.003–0.049)	0.008 (0.002–0.038)	

	*Home remedy use*				
	Yes (%)	No (%)			
*Faculty*					**0.510**
Nonmedical	180 (46.3)	113 (29.0)	1	1	
Medical (health)	57 (14.7)	39 (10.0)	1.090 (0.681–1.744)	1.198 (0.700–2.052)	
*Degree of menstrual pain*					0.000^**∗**^
No pain	13 (3.3)	68 (17.5)	1	1	
Mild pain	53 (13.6)	36 (9.3)	0.130 (0.063–0269)	0.0128 (0.061–0.265)	
Moderate pain	101 (26)	36 (9.3)	0.068 (0.034–0.138)	0.067 (0.033–0.137)	
Sever pain	70 (18)	12 (3.1)	0.033 (0.014–0.077)	0.033 (0.014–0.077)	

^*∗*^ *P* value less than 0.05.

## Abbreviations

CMHS: College of Medicine and Health Sciences
SPSS: Statistical Packages for Social Sciences.
